# Editorial: Causal associations between nutrition, health, and genes: an evidence-based approach

**DOI:** 10.3389/fnut.2025.1592666

**Published:** 2025-04-08

**Authors:** Kristen J. Sutton, Lauren O'Connor, Cheng Zheng, Nicola Pirastu, Joanne B. Cole

**Affiliations:** ^1^Department of Biomedical Informatics, University of Colorado Anschutz Medical Campus, Aurora, CO, United States; ^2^Beltsville Human Nutrition Research Center, Agricultural Research Service, United States Department of Agriculture, Beltsville, MD, United States; ^3^Department of Biostatistics, University of Nebraska Medical Center, Omaha, NE, United States; ^4^Human Technopole, Fondazione Human Technopole, Milan, Italy

**Keywords:** nutrigenomics, Mendelian randomization, causal inference, randomized controlled trial, nutritional epidemiology

Understanding the intricate interplay between nutrition, genes, and health is paramount with chronic diseases on the rise and personalized medicine and precision nutrition gaining ground. However, it is difficult to establish causality in this field because of ethical and practical limitations precluding randomized controlled trials (RCTs). Mendelian randomization (MR) is a useful causal inference method that uses a genetic instrument (GI) to estimate the effect of exposures, such as dietary intake, on health-related outcomes. For an MR analysis to be valid, the GI must meet three statistical assumptions including (1) relevance—the GI is associated with the exposure; (2) exchangeability—there are no confounders of the GI and the outcome; and (3) exclusion restriction—the GI is related to the outcome only through the exposure. In this Research Topic we highlight a collection of manuscripts that use causal inference methods, including MR and RCTs, to unravel the complex relationships between our genes, dietary choices, and disease risk, including cancer, cardiovascular disease, and digestive diseases and disorders.

The manuscripts included in this Research Topic underscore the importance of modeling critical confounders that may influence MR results. For example, Liu and Cai investigated the relationship of cereal intake on seven types of cardiovascular disease (CVD) using univariable and multivariable MR (MVMR). In univariable MR, several protective relationships were identified between different cereal types and CVD. However, the authors explored the direct effects of cereals on CVD while controlling for education or income using MVMR due to the potential role of socioeconomic factors (SES) influencing dietary intake. They found that all relationships were attenuated except for muesli, a granola and dried fruit-based cereal, on heart failure. Two other studies employed two-sample MR to evaluate the impact of metabolites on kidney function. Shao et al. evaluated 100 circulating plasma metabolites and 263 drugs on nephritis and Liu et al. evaluated over 480 metabolites on chronic kidney disease (CKD) and creatinine-based estimated glomerular filtration rate (eGFR). In the latter article, causal relationships were inferred for betaine and N-acetylornithine on kidney function. However, upon performing MVMR, the investigators only addressed whether the metabolites acted on kidney function independently. Future research on this topic should consider employing MVMR to quantify both the direct and indirect effects of potential confounders such as health diagnoses, SES, and other disease biomarkers.

Aside from confounders, researchers should also carefully consider effect modifiers and population characteristics when modeling the causal relationships among variables in MR. For example, in the study by Astore and Gibson they combined non-genetic and genetic analyses to evaluate the relationship between omega-3, omega-6, and docosahexaenoic acid (DHA) on over 1,200 diseases. Of the relationships identified (ranging from 170 for omega-3 to 285 for DHA) almost all were in the direction of disease protection. The authors then evaluated whether these factors were mediated by obesity and found that obesity interacts with omega-3 in a disease-specific manner, serving a protective role in some disease states while being neutral in others. The identification of nutrient-disease interactions, such as these illustrates how health status is important to consider when evaluating the effect of dietary-derived factors on health.

The relationship between micronutrients and pregnancy complications was explored by Xie et al.. Of calcium, phosphorus, magnesium, iron, zinc, copper, vitamin A, B6, B12, C, D, E, nicotinamide, and folate only one relationship between Vitamin E and spontaneous abortion was identified but failed to replicate in an independent cohort. The authors point out a limitation that the micronutrient exposures were obtained from sex-combined GWAS, whereas the outcomes were female specific. This draws attention to the need for more sex-aware GWAS to enable sex-aware MR analyses and further highlights the importance of considering population characteristics.

The role of coffee and coffee related metabolites including caffeine, theobromine, theophylline and paraxanthine on brain structures and volume was evaluated using two-sample MR by Luo et al.. Theobromine was causally associated with the cerebral-cortex surface area and hippocampus volume. Demonstrating that metabolites of coffee may impact brain characteristics, the authors explored the relationships among the brain structures, metabolites and neurological diseases such as depression and Parkinson's using a triangulation approach and suggested these factors may be related.

The final manuscripts in the Research Topic focused on digestive diseases and disorders. The first led, by Zhang et al., used two-sample MR to evaluate fifteen blood cell traits on ulcerative colitis and inflammatory bowel disease and found that eosinophils, a type of white blood cell involved in the immune system, may affect ulcerative colitis. The second, led by Mazhar et al., performed a randomized, double blinded, placebo-controlled to investigate the impact of a dietary enzyme supplement on macromolecule digestion in samples from participants with an ileostomy from a previous small bowel resection. The supplement increased monosaccharide levels suggesting it could accelerate food digestion and may increase nutrient availability. These two articles highlight how implementing different types of causal research designs can help move the field forward in different but meaningful ways.

Overall, this Research Topic illustrates how causal methods including MR and RCTs can help elucidate causal relationships between foods, beverages, and nutrients on health outcomes. While RCTs are considered the gold-standard method for testing causality, MR serves as an important and complementary method in the causal toolkit. For example, the low barriers of entry allow MR to play a discovery role identifying new potentially causal relationships, which can serve as hypotheses to test in RCTs with a high likelihood of success. When RCTs are not possible, MR can also be used to study exposures that cannot be tested ethically such as alcohol intake.

This Research Topic also brings to light some concerns. The availability of GWAS summary statistics and MR software make these analyses perhaps too easy to conduct without thorough investigation. For example, the exclusion restriction assumption is violated if the GI is associated with traits that affect the outcome through paths other than the exposure (e.g., horizontal pleiotropy). Several strategies have been developed to identify and/or address pleiotropy, including the MR-Egger intercept test, MR-PRESSO outlier test, removing variants associated with confounding traits using phenome-wide scans, or accounting for the direct and indirect effects of dietary intake while controlling for confounders using MVMR; yet, these steps are not consistently completed. The importance of accounting for GI specificity was demonstrated by Liu and Cai in their evaluation of cereal on CVD. When they accounted for the effects of education and income using MVMR, most causal associations between cereal and CVD were attenuated. Another gap was highlighted by Xie et al. who performed analyses of micronutrient status on pregnancy complications but used sex-combined GWAS for their exposures due to the unavailability of sex-aware summary statistics. More sex-aware GWAS are needed to improve the precision of MR estimates when the exposure or outcome are suspected to be affected by sex.

In sum, while RCTs are considered the gold-standard for assessing causality, MR is an important tool that can help prioritize resources for RCTs. Continued attention to GI mis-specificity and persistent pleiotropy is vital for identifying the true underlying and un-confounded causal relationships between dietary factors and human health.

